# An outbreak of trichomonosis in European greenfinches *Chloris chloris* and European goldfinches *Carduelis carduelis* wintering in Northern France

**DOI:** 10.1051/parasite/2019022

**Published:** 2019-04-08

**Authors:** Jean-Marc Chavatte, Philippe Giraud, Delphine Esperet, Grégory Place, François Cavalier, Irène Landau

**Affiliations:** 1 UMR 7245 MCAM MNHN CNRS, Muséum National d’Histoire Naturelle 61 rue Buffon, CP52 75231 Paris Cedex 05 France; 2 Cap-Ornis Baguage 10 Rue de la Maladrerie 59181 Steenwerck France; 3 Laboratoire Départemental d’Analyse du Pas-de-Calais (LDA 62) 2 Rue du Genévrier, SP18 62022 Arras Cedex France; 4 Laboratoire Labéo Manche 1352 Avenue de Paris, CS 33608 50008 Saint-Lô Cedex France

**Keywords:** *Trichomonas gallinae*, Finch trichomonosis, Outbreak, France, Wintering birds

## Abstract

Avian trichomonosis is a common and widespread disease, traditionally affecting columbids and raptors, and recently emerging among finch populations mainly in Europe. Across Europe, finch trichomonosis is caused by a single clonal strain of *Trichomonas gallinae* and negatively impacts finch populations. Here, we report an outbreak of finch trichomonosis in the wintering populations of *Chloris chloris* (European greenfinch) and *Carduelis carduelis* (European goldfinch) from the Boulonnais, in northern France. The outbreak was detected and monitored by bird ringers during their wintering bird ringing protocols. A total of 105 records from 12 sites were collected during the first quarter of 2017, with 46 and 59 concerning dead and diseased birds, respectively. Fourteen carcasses from two locations were necropsied and screened for multiple pathogens; the only causative agent identified was *T. gallinae*. Genetic characterization was performed by four markers (small subunit ribosomal RNA, hydrogenosomal iron-hydrogenase, and RNA polymerase II subunit 1 genes, and the internal transcribed spacers (ITS) region) and confirmed the *T. gallinae* strain to be A1, which affects the finch populations of Europe. This was also confirmed by an ITS-based phylogenetic analysis which further illustrated the diversity of the *Trichomonas* infecting birds. Preliminary data on the survival and dispersion of infected birds were obtained from ring-returns of diseased individuals. The anthropogenic spread of diseases through bird feeding practices is highlighted and some suggestions to prevent pathogen transmission via backyard supplementary feeders for garden birds are given.

## Abstract (fr)

La trichomonose aviaire est une maladie commune et largement répandue chez les columbidés et les rapaces, devenant émergente au sein des populations de fringillidés, essentiellement en Europe. Dans l’Europe entière, la trichomonose des fringillidés est causée par une souche unique de *Trichomonas gallinae*, ayant un impact néfaste sur leurs populations. Cette étude décrit une épidémie de trichomonose des fringillidés au sein des populations de *Chloris chloris* (verdiers d’Europe) et de *Carduelis carduelis* (chardonnerets élégants) hivernantes dans le Boulonnais dans le nord de la France. L’épidémie fut détectée et suivie par les bagueurs durant leurs programmes de baguage des oiseaux hivernants. Un total de 105 signalements comptabilisant 46 oiseaux morts et 59 oiseaux montrant des signes cliniques furent collectés au cours du premier trimestre 2017. Quatorze carcasses provenant de deux localités furent autopsiées pour la recherche d’agents pathogènes et seul *T. gallinae* fut mis en cause. Une caractérisation génétique de la souche de *T. gallinae* impliquée fut réalisée à l’aide de 4 marqueurs moléculaires, les gènes de l’ARN ribosomique 18S, de l’hydrogénase à fer hydrogénosomale et de l’ARN polymérase II, et la région des espaceurs internes transcrits (ITS) et confirma qu’il s’agissait de la souche A1 qui affecte les populations de fringillidés d’Europe. Ce résultat fut confirmé par une analyse phylogénétique basée sur les ITS qui, de plus, illustra la diversité des *Trichomonas* infectant les oiseaux. Les retours de bagues ont permis la collecte de données préliminaires sur la survie et la dispersion des oiseaux infectés bagués. La dissémination de maladies en lien avec les activités anthropogéniques, telles que le nourrissage des oiseaux, est mis en évidence et des suggestions sont proposées pour limiter les risques de transmission d’agents pathogènes par les mangeoires.

## Introduction

Avian trichomonosis is caused by the flagellate protozoan *Trichomonas gallinae* (Rivolta, 1878) [74, 79]. This parasite has a worldwide distribution and primarily infects Columbiformes [11, 35, 38, 55, 58, 72, 74–76, 79, 81] and raptors [6, 11, 12, 37, 40, 42, 67, 71, 72, 77, 79] in which it can cause a disease known as canker and frounce, respectively [5, 8, 77]. Depending of the pathogenicity of the strain as well as the immune status of the bird host, the infection might remain benign or progress to severe infection [11, 12, 58, 75, 76, 81], sometimes with high fatality rates [19, 24, 31, 35, 38]. In Columbiformes, nearly all individuals are carrier of trichomonads and canker is a common disease of domestic, racing and wild pigeons with a high rate of infection in *Columba livia* that can act as carriers and reservoirs [24]. Aside from the Columbiformes and the three orders of birds of prey, this parasite has also been found in Galliformes [59, 63], Psittaciformes [4, 55], Struthioniformes [66], Passeriformes [8, 20, 24], Gruiformes [24], Anseriformes [24] and Piciformes [20].

Finch trichomonosis was known to be possible from experimental infection [78, 79] and has been infrequently reported from captive and free-ranging finches [2, 8]. It has become widely known since 2005 following an epidemic that affected essentially European greenfinches *Chloris chloris* (Linnaeus, 1758) and Common chaffinches *Fringilla coelebs* Linnaeus, 1758 in the West of the United Kingdom [13, 39, 64]. The following years, seasonal epidemic mortality occurred in the UK in late summer/autumn [44, 70] and spread to the eastern UK in 2007 [47, 70]. This emerging disease subsequently rapidly spread to southern Fennoscandia in 2008 [46, 61], northern Germany in 2009 [65], central and western France with suspected and confirmed cases in 2010 and 2011, respectively [32, 33], then Austria and Slovenia in 2012 [27, 83]. Based on the disease spreading pattern and historical ring returns, the spread of disease is likely attributable to migrating birds and suggested the Common chaffinches as the primary vector [46]. Finch trichomonosis has adversely impacted the *C. chloris* and *F. coelebs* populations [47, 48, 50, 70], e.g., in the UK, an estimated reduction of up to 66% of the breeding population of European greenfinches has been observed with a continuous decline since the emergence of the disease [48], while no reduction has been noted in the breeding populations of Common chaffinches; in southern Finland, a significant declines of 47% in breeding numbers and 65% in wintering numbers for European greenfinches, and 4% for the breeding number of Common chaffinches [50]. At the same time that the disease has spread across Europe, finch trichomonosis has also been reported in small numbers of individuals from the regions of eastern Canada since 2007 [25, 26].

In Europe, while the disease mainly affects *C. chloris* and *F. coelebs*, several other fringillids such as *Carduelis carduelis* (Linnaeus, 1758), Siskin *Spinus spinus* (Linnaeus, 1758), Hawfinch *Coccothraustes coccothraustes* (Linnaeus, 1758), Bullfinch *Pyrrhula pyrrhula* (Linnaeus, 1758), Brambling *Fringilla montifringilla* Linnaeus, 1758, as well as other garden birds, namely House sparrow *Passer domesticus* (Linnaeus, 1758), Yellowhammer *Emberiza citronella* Linnaeus, 1758, Dunnock *Prunella modularis* (Linnaeus, 1758) and Great tit *Parus major* Linnaeus, 1758 were also found to be infected [70] or diseased and dead [32, 83] but in small numbers. In the Canadian Maritime provinces, diseased and dead bird species were essentially the Purple finch *Haemorhous purpureus* (Gmelin, 1789) and the American goldfinch *Spinus tristis* (Linnaeus, 1758) [25, 26].

*Trichomonas gallinae* primarily infects the upper digestive tract in the pharyngeal form but may also develop in a visceral form with infection of the internal organs [11, 60, 74, 79]. In the classic pharyngeal form, the pathogenic strains induce fibronecrotic oesophagitis and ingluvitis which start with whitish to yellowish limited lesions that progressively grow into large caseous masses that obstruct the lumen of the pharynx, oesophagus and crop. These lesions limit or totally prevent food and water intake and therefore impair breath and lead to starvation, suffocation, apathy, weakening, emaciation and death [11, 58, 74, 79]. The affected birds classically show signs of general illness, reduced body conditions, lethargy, ruffled plumage and no fleeing behaviour. In addition, they classically display sialism that impairs breath (Supplementary File – Video) and regurgitate food that wets plumage around the face and beak, and also show signs of breathlessness. Secondary infection by bacteria may occur and worsens the clinical situation. The visceral form almost ineluctably leads to death [60, 79]. The progress of the disease may extend over several days or even weeks [79].

Since *T. gallinae* has no cyst form, the route of transmission is by direct contamination. In the Columbiformes, the parasite is transmitted to the brood immediately after hatching from the crop milk [11, 79]. Raptors become infected when eating infected prey [11, 73]. The other birds are infected from water and food contaminated by the saliva of infected birds. This route is likely the most common among the farm and domestic birds, game birds, as well as garden birds through feeders, troughs and baths [2, 38, 41, 54].

Results of the genetic characterization performed on the internal transcribed spacer region (ITS region) and 18S small subunit ribosomal RNA (18S ssrRNA) gene from *T. gallinae* strains causing finch trichomonosis in the UK and Fennoscandia were found to be similar and suggest that only one strain of *T. gallinae* is the causative agent of emerging finch trichomonosis [45, 46]. Further genetic characterization performed on samples from the UK, Austria and Slovenia, using additional targets such as iron-hydrogenase and RNA polymerase II genes, has confirmed the clonality of the *T. gallinae* strain involved [27].

In the context of emerging finch trichomonosis among European fringillid populations, the present study reports the epidemiological monitoring of a large outbreak of this diseases that occurred in France, in the Boulonnais region during winter 2016/2017, and provides genetic characterization of the *T. gallinae* strain involved.

## Materials and methods

### Epidemiological data

During winter 2016/2017, a total of 105 records about dead or diseased birds essentially *C. chloris* and *C. carduelis* were collected in the first quarter of 2017. In all, 97 reports originated from bird ringers working with the Association Cap-Ornis Baguage, Steenwerck, Pas-de-Calais (62), France during their winter monitoring through the SPOL-Mangeoire protocol (a standardised protocol to study wintering strategies of the common passerines in France at provisioned feeding stations) [36]; one originated from the Regional Centre for Wildlife Healthcare, Moncavrel, France; and seven from individuals who reported to the Research Centre on the Biology of Bird Populations (CRBPO), National Museum of Natural History (MNHN), Paris, France. Among these data, the ring returns accounted for 14 records that provided information about survival of the infected birds as well as further illustrated their dispersion movements for seven of them. Duplicate records for birds noted sick and subsequently found dead may be possible but were ruled out for the sick ringed individuals.

### Geographical area

**Figure 1:**
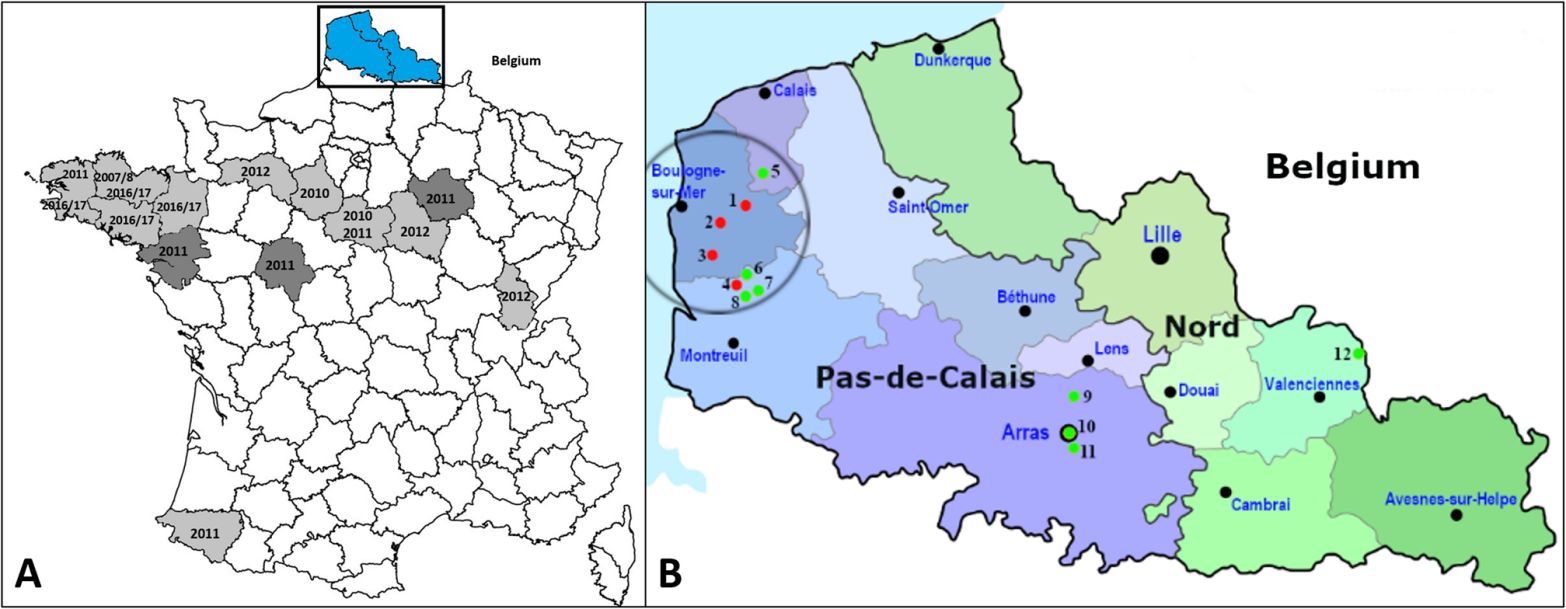
General map illustrating previous reports of finch trichomonosis in France and detailed map of northern France illustrating the geographical origins of the data about dead and diseased birds. (**A**) Administrative départements with confirmed and suspected events of finch trichomonosis in dark and light grey, respectively; Départements in blue represent the northern France area and contains the studied sites; Years of the events are written for each département, years with a slash represent winter event. (**B**) The circle broadly defines the Boulonnais region. Red dots represent bird ringing stations within the Boulonnais where dead and diseased birds where observed and recorded over the first trimester of 2017 with 1: Bournonville, 2: Wirwignes, 3: Tingry and 4: Parenty; Green dots represent additional locations where dead birds were retrieved with 5: Herbinghen, 6: Bezinghem, 7: Preures, 8: Enquin-sur-Baillons in the Boulonnais, and 9: Vimy, 10: Arras, 11: Achicourt and 12: Saint-Aybert in the inland area.

All the data concerning dead or diseased birds included in this report originated from the Nord–Pas-de-Calais Region in northern France (Fig. 1A and B), with 101 records from eight sites in the Boulonnais area, namely Bezinghem, Bournonville, Enquin-sur-Baillons, Herbinghen, Parenty, Preures, Tingry and Wirwignes; and four additional records from inland sites, namely Achicourt, Arras, Saint-Aybert and Vimy (Fig. 1B).

### Biological material

A total of 14 fresh carcasses (<24 h death) were collected in February 2017 from Parenty (five *C. chloris*, stored at 4 °C) and Bournonville (five *C. chloris*, two *C. carduelis*, one *F. coelebs* and one *P. modularis*, stored at −20 °C) by G. Place and F. Cavalier, respectively. They were reported to the SAGIR network, a wildlife disease epidemiological and surveillance network [17] of the National Hunting and Wildlife Agency (ONCFS).

### Laboratory tests and procedures

The carcasses were sent for diagnosis and testing to the Laboratoire Départemental d’Analyse du Pas-de-Calais (LDA 62) which performed the necropsies, the macroscopic anatomo-pathologic examinations, the bacteriology and coprology testing, as well as the sampling for the histology and molecular tests, according to recommended procedures [28]. Bacteriology testing was performed to detect the presence of *Salmonella* according to a standard protocol [62] and other potential pathogenic bacteria from lesions and internal organs, including at least the liver, spleen and brain. Coprology was performed by direct examination of scrapings from three levels of the digestive tract [21]. Tissue samples taken from the birds from Parenty were transferred to Laboratoire Vet Diagnostics, Lyon, France, for histological investigations. Based on the epidemiological context and lesions observed, LDA 62 collected samples for certain specific pathogens to be tested by molecular methods such as: (i) tracheal and rectal swabs (Virocult^®^) for Avian Influenza Virus (AIV) tested by an accredited laboratory (Laboratoire Inovalys, Nantes, France) according to the protocol: rRT-PCR AIV H5-HA2 with IPC-M [3]; (ii) tissue samples for the Usutu Virus (USUV) tested by the reference laboratory (ANSES, Maisons-Alfort, France) according to the protocol of [49]; and (iii) dry pharyngo-laryngeal swabs preserved in 70% ethanol for *Trichomonas*, forwarded to Laboratoire Labéo Manche, Saint-Lô, France and tested according to [69].

### Additional molecular tests for *T. gallinae*

DNA extracted in Laboratoire Labéo Manche were plotted onto FTA cards and transferred to the Parasitology Laboratory of the MNHN. Confirmation and additional molecular investigations by sequencing and genotyping of the involved strain of *T. gallinae* were obtained using four markers, namely the 18S ssrRNA gene, the ITS region, the hydrogenosomal iron-hydrogenase (hdg) gene and the RNA polymerase II subunit 1 (rpb1) gene. The 18S ssrRNA gene and ITS region were amplified together in a combined PCR using the forward and reverse oligonucleotide primers developed by [56] and [23], respectively. The hdg and rpb1 gene fragment amplifications were performed according the protocols developed by [45] and [27], respectively. PCRs, visualization of the PCR products by agarose gel electrophoresis, gel purification and storage of the purified products were performed as described previously [9]. Bidirectional sequencing was prepared with a BigDye^®^ Terminator v3.1 cycle sequencing kit (Applied Biosystems^®^) with the oligonucleotide primers used for the amplification and four internal oligonucleotides primers, namely Medlin-R [56]; 1055F and 1055R [7], and 564R in the forward direction [82]. Purification of the BigDye^®^ reaction products, bi-directional sequencing, alignment, visual cross-checking of the fragments sequenced, and then generation of the consensus sequences were performed as described previously [9].

Comparison of each sequence was done by basic local alignment search tool (BLAST) [1].

### Phylogenetic analysis

For the ITS region, multiple alignment of the sequences obtained for *T. gallinae* and 53 sequences of *Trichomonas* retrieved from GenBank was generated using the Clustal W algorithm [43]. Molecular phylogeny was inferred by the maximum likelihood (ML) method based on the Tamura 3-parameter + Γ + I model of evolution [80] with the most appropriate model of nucleotide substitution for ML analysis chosen using the Bayesian Information Criterion score [73]. Nodal robustness and reliability of inferred trees topologies were assessed by non-parametric bootstrap analysis using 1000 replicates. Initial trees for the heuristic search were obtained automatically by applying NJ and BioNJ algorithms to a matrix of pairwise distances estimated using the Maximum Composite Likelihood approach, and then selecting the topology with superior log likelihood value.

## Results

### Epidemiological monitoring

**Figure 2:**
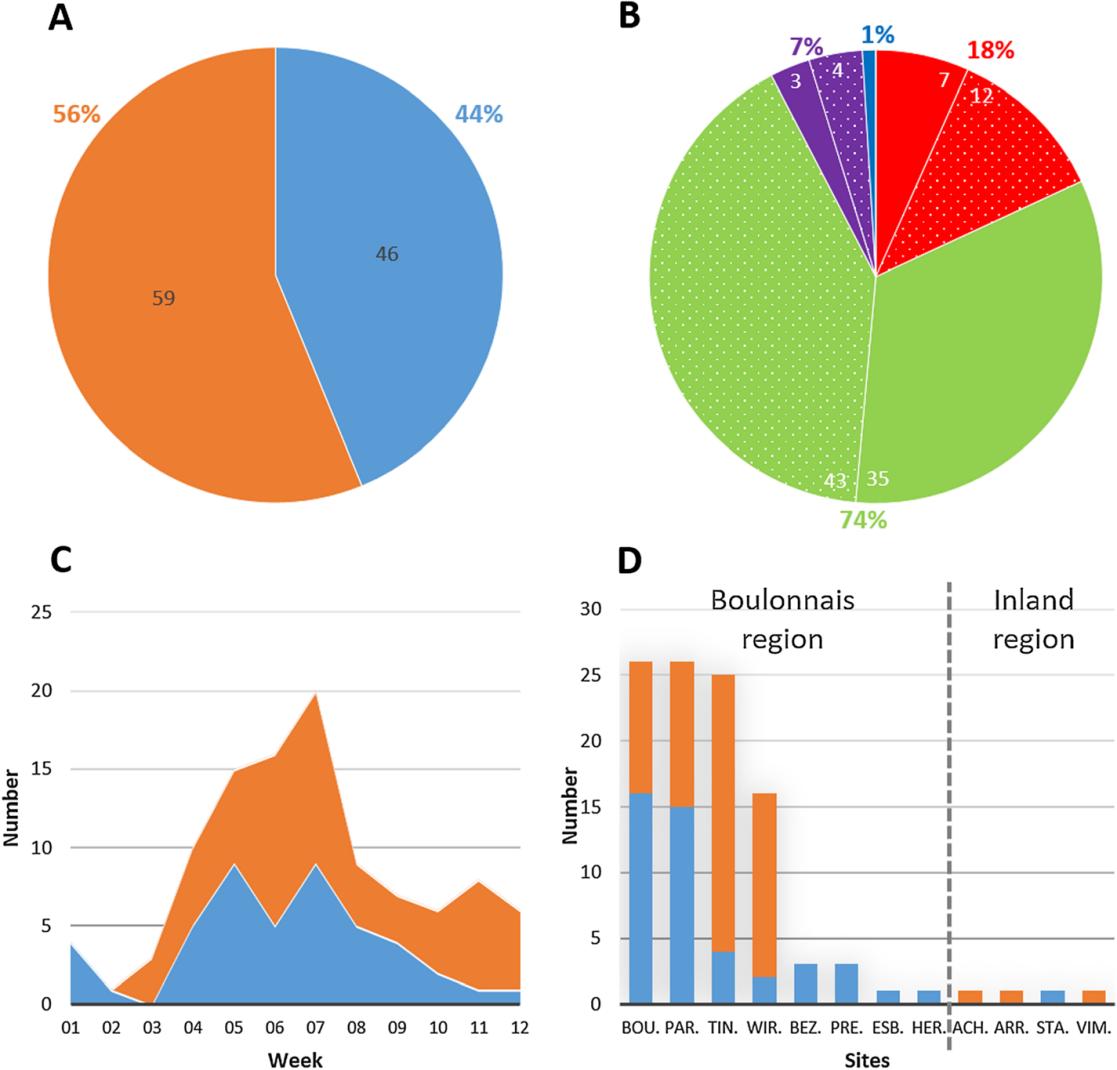
Epidemiological data about the outbreak of finch trichomonosis from the Boulonnais during the first quarter of 2017. (**A**) Number and percentage of dead and diseased bird records (*n* = 105). (**B**) Species distribution of dead (solid) and diseased (dotted) bird records with green, red, purple and blue representing European greenfinch, European goldfinch, Common chaffinches and Dunnock, respectively (*n* = 105). (**C**) Weekly changes in the number of dead and diseased bird records (*n* = 105). (**D**) geographical distribution of dead and diseased bird records (*n* = 105); Bou: Bournonville, Par: Parenty, Tin: Tingry, Wir: Wirwignes, Bez: Bezinghem, Pre: Preures, EsB: Enquin-sur-Baillons, Her: Herbinghen, Ach: Achicourt, Arr: Arras, StA: Saint-Aybert and Vim: Vimy. In A,C,D blue and orange represent dead and diseased birds, respectively.

A total of 105 records were collected during the first quarter of the year 2017, with 46 (44%) dead and 59 (56%) diseased birds, respectively (Fig. 2A). The records originated from four bird species, with the European greenfinch being the most affected (74%) followed by the European goldfinch (18%), the Common chaffinches (7%), and the Dunnock (1%) (Fig. 2B). To monitor the progress of the outbreak, the records of dead and diseased birds were weekly split (Fig. 2C). The outbreak was quite sudden as prior to it only two records of dead *C. chloris* had been noted by the bird ringers in the Boulonnais region since October and were related to predation (pers. comm.). The first suspicious fatalities that triggered an alert among the bird ringers were recorded in 2017, weeks 01 and 02. They were followed by a sharp rise in the number of records of both diseased and dead birds from week 03 to reach a peak in week 07. Then a decrease in the number of records was noted to a stabilisation from week 09 onward (Fig. 2C). As wintering bird ringing protocols [36] usually end with the start of pre-nuptial migration and the breeding season, the monitoring ceased in week 12. The geographical distribution clearly showed a focus of infection in the Boulonnais region with 101 (96.2%) records of dead and diseased birds from eight sites (Fig. 2D).

### Ringed bird data

Among the records of a total of 26 birds which carried rings, 15 provided some information. Nine birds were ringed in healthy conditions before the outbreak as early as Feb 2015 and might represent local breeders or migrants that wintered in the same area. Among them, seven were found dead during the outbreak between 0 and 8.5 km from their birding sites (Fig. 1B), while the remaining two were recaptured and noted diseased during the outbreak. Seventeen diseased birds were ringed during the outbreak. Among them six were found dead between 0 and 5 days after ringing and between 0 and 4 km from their capture sites (Fig. 1B). Considering the significant likelihood of underreported carcasses, the observed recovery rate of ringed sick individuals, 35.3% (6/17) is a very low figure and the actual death rate must have been much higher. The numbers are small, but as expected based on the characteristic clinical manifestations of trichomonosis, tend to indicate that diseased birds neither survive for a long time, nor move far from feeders.

### Necropsies and histopathological investigations

**Figure 3:**
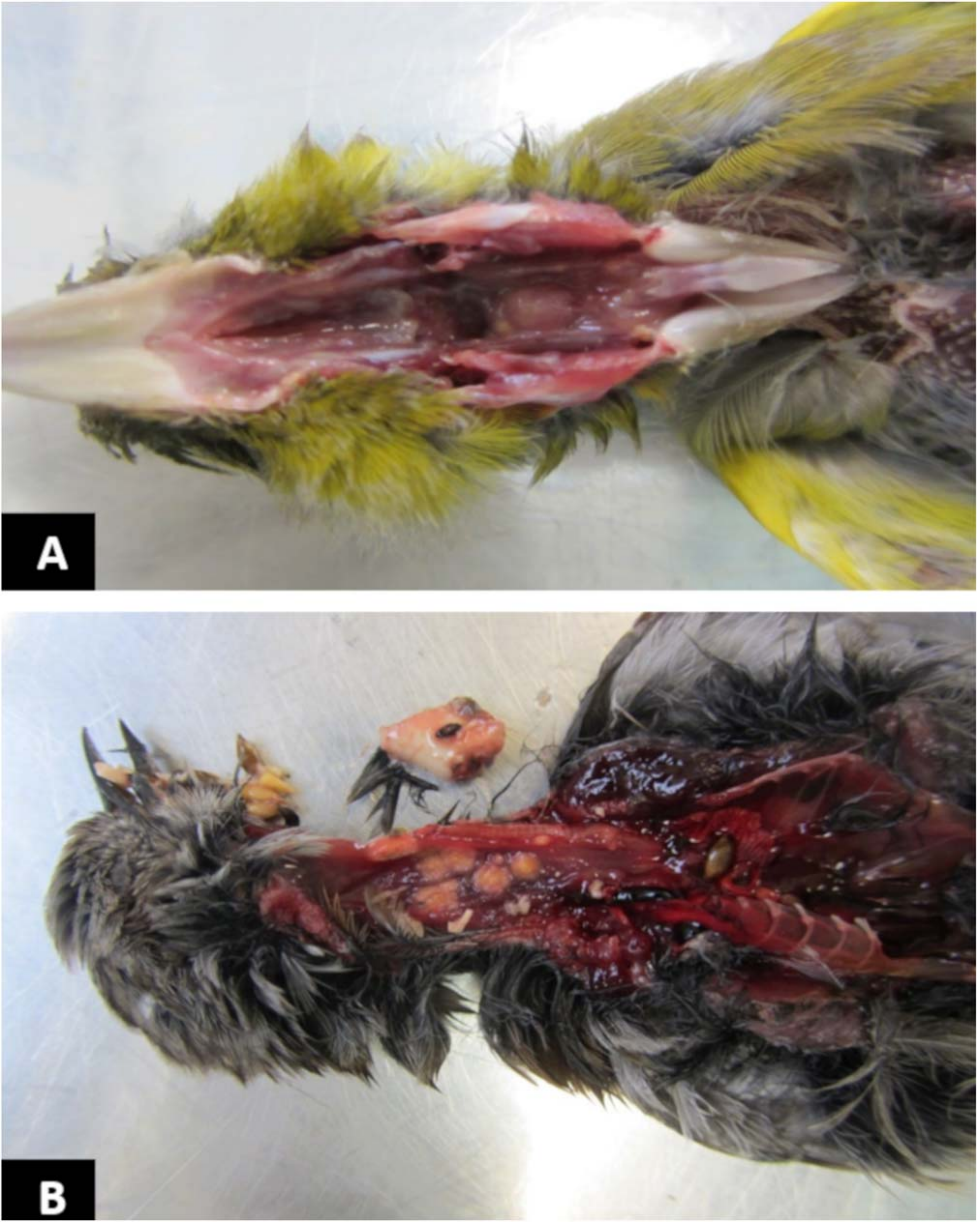
Photographs of the gross lesions observed during necropsies of the carcasses. (**A**) European greenfinch from Parenty presented unspecific small yellowish, adherent and mucoid pharyngeal lesions. (**B**) Dunnock from Bournonville presented characteristic oesopharyngeal fibrino-necrotic lesions of avian trichomonosis. Photos P. Giraud.

All five carcasses from Parenty showed non-specific moderate enteritis combined for three individuals with small yellowish, adherent and mucoid formations in the pharynx (Fig. 3A). Based on histology, these formations are caused by hyperkeratosis and hyperplasia of the oral mucosa and were sporadically observed with trichomonosis. All the carcasses from Bournonville revealed the presence of fibrino-necrotic lesions in the oesopharyngeal region compatible with infection by *Trichomonas*. These lesions were particularly prominent and extended in four carcasses (one *C. chloris*, one *C. carduelis*, one *F. coelebs* and one *P. modularis*) (Fig. 3B). Unfortunately, neither the histological preparations nor the lesion imprints allowed direct observation of trophozoites of *T. gallinae*. No parasite culture was initiated but swabs from the lesions were collected for molecular biological testing.

### Investigations for infectious diseases

The coprological examinations were negative. The bacteriological tests did not detect *Salmonella* nor common pathological bacteria (e.g., *Listeria*, *Yersinia*, *Pasteurella* among others) from the carcasses. Similarly, no viral infections by AIV or USUV were detected by the specific PCRs. Parasitic infection by *T. gallinae* was tested by PCR and DNA and positive samples were successfully detected in the pool of swabs from Parenty and from four individual swabs (three *C. chloris*, one *C. carduelis*) from Bournonville. The swabs from the *F. coelebs* and *P. modularis* were repeated and remained negative despite the presence of compatible lesions.

### Molecular confirmation and subtyping

All five positive *T. gallinae* DNAs were double confirmed by PCR amplification and sequencing of a large DNA fragment that combined the 18S ssrRNA gene and ITS region. Additional genotyping was successfully conducted using the hdg and the rpb1 genes. All five samples were identical for all four genetic targets and confirm a single clonal strain involved. The sequencing of these four genetic targets lead to three fragments of 1924 bp, 1033 bp and 1437 bp for the 18S ssrRNA + ITS region together, the hdg and the rpb1 genes, respectively and are deposited in GenBank under accession numbers MK172843–MK172857.

The BLAST comparison showed 100% identity with other *T. gallinae* sequences deposited in GenBank for all four genetic targets (Supplementary File). The Boulonnais epidemic strain can be classified as genotype A/B or genotype IV based on the ITS region according to the nomenclatures of Gerhold et al. [29] and Grabensteiner et al. [34] respectively; genotype IV based on the 18S ssrRNA gene according to the nomenclature of Grabensteiner et al. [34]; and further classified as subtype A1 based on the hdg gene according to the nomenclature of Chi et al. [10]. These classifications all designate the same *T. gallinae* strain recovered worldwide from a wide range of bird hosts, and this strain is known to be responsible for finch trichomonosis in the UK and across Europe (Supplementary File). This result was further confirmed by the additional genetic comparison based on the rpb1 genes which reported 100% identity with the sequences of this pathogenic *T. gallinae* strain (Supplementary File).

### Phylogenetic analysis

**Figure 4:**
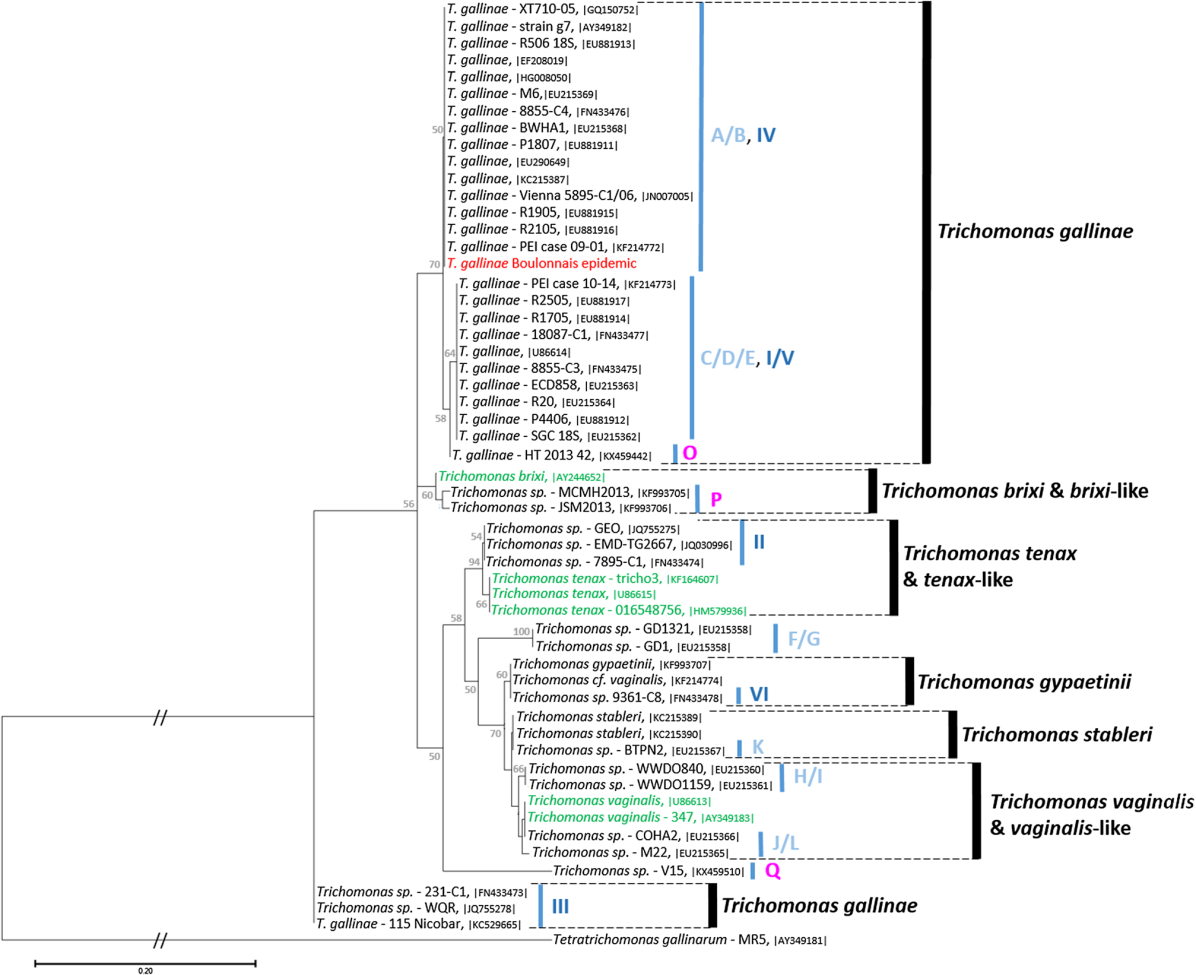
Molecular phylogeny of avian *Trichomonas* and related species inferred on the internal transcribed spacer (ITS) region. The analysis was performed by Maximum Likelihood (ML) with the Tamura 3-parameter + Γ + I model of evolution [78] on a 371 bp fragment covering the ITS1–5.8SrRNA–ITS2 region of our sequences MK172843 in red, 47 sequences of avian *Trichomonas* in black, six related sequences of *Trichomonas* infecting mammals in green and *Tetratrichomonas gallinarum* as the outgroup to root the tree. Accession numbers of the sequences are provided between vertical bars. The reliability of tree topologies, nodal robustness, and statistical branch support were assessed by non-parametric bootstrap analysis using 1000 replicates (only >50% shown). Avian *Trichomonas* genotype names according to the classification of Gerhold et al. [29] and Grabsteinner et al. [34] are given in light and dark blue, respectively. Recent avian *Trichomonas* genotypes reported by Marx et al. [52] are given in purple.

In the phylogenetic analysis based on the ITS region, the Boulonnais epidemic strain clusters with *T. gallinae* genotype A/B or genotype IV according to the nomenclatures of Gerhold et al. [29] and Grabensteiner et al. [34] respectively (Fig. 4). Like in many other studies [10, 29, 30, 34, 51, 52, 72], this analysis also highlights the diversity of avian *Trichomonas* which present a multitude of genotypes. Two of them have recently been recognised as distinct species, namely *Trichomonas stableri* Girard et al., 2014 [30] and *Trichomonas gypaetinii* Martínez-Díaz et al., 2015 [51]; several others are related to *Trichomonas* species of Mammals such as *Trichomonas tenax*, *Trichomonas brixi* and also *Trichomonas vaginalis*; while some are unrelated to any described species (e.g., genotypes F/G or genotype Q according to the nomenclatures of Gerhold et al. [29] and Marx et al. [52], respectively) and might constitute distinct species. Then, in this analysis, *T. gallinae* appears paraphyletic as some sequences of *Trichomonas gallinae* isolated from several pigeon species from Europe and Australia, grouped under genotype III according to the nomenclature of Grabensteiner et al. [34] form a totally separate clade that branch out early at the base of the phylogenetic tree (Fig. 4).

## Discussion

### Usefulness of molecular methods in the diagnosis and characterization of avian *Trichomonas*

In the Boulonnais outbreak, based on the clinical signs (reduced body conditions, lethargy, ruffled plumage, sialism, breathlessness, wet plumage around the face and beak) and the observation of typical gross lesions in some carcasses at the time of the necropsies, the diagnosis of trichomonosis was likely. However, no confirmative observation of trophozoites of *Trichomonas* was possible, hampering the possibility of specific identification by morphology. For small birds, this is unfortunately often the case due to the rapid decaying process of the carcass, leading to suspicion without confirmation about the nature of the causative agent [14–16, 32, 53]. Even in a more fortunate situation with observable trophozoites, the identification of *Trichomonas* parasites at the species level remains challenging since *T. gallinae* display great morphological variability and also represent the high diversity of *Trichomonas* species recovered from birds [30, 51, 57]. Therefore, the use of molecular methods was extremely valuable, firstly to confirm *Trichomonas* infection, secondly to identify the species as *T. gallinae* and thirdly to characterize the particular pathogenic strain causing the outbreak [2, 10, 27, 29–32, 34, 45–47, 52, 70, 72, 83].

### Occurrences of finch trichomonosis in France

While a survey for columbid trichomonosis has been in place in France since 2010, following some unusual mortalities in Stock dove chicks (*Columba oenas* Linnaeus, 1758) from 2008 to 2010 [15], no specific surveillance was set up for finch trichomonosis despite the emergence and spread of the disease in the UK.

The first report about finch trichomonosis in France appeared as a simple mention without detail related to an episode of mortality of *C. chloris* during winter 2007/2008 in the Côtes-d’Armor that could have apparently been caused by *Trichomonas* [14, 53, 68] (Fig. 1A). Another suspicion of finch trichomonosis based on typical gross lesions was reported in May–June 2010 from an outbreak in Eure-et-Loire involving 20 *C. chloris*, two *C. carduelis* and one *P. domesticus*; and in July 2010 from Loiret [15, 32] (Fig. 1A). Then, finch trichomonosis was confirmed in France in April–June 2011 based on laboratory results from an outbreak from Loiret involving 20 *C. chloris* and a mortality of 15 *C. chloris* from Indre-et-Loire following massive feeding with sunflower seeds [16, 32] (Fig. 1A). The same year, *F. coelebs* mortality concomitantly with columbids and caused by *Trichomonas* was reported in Pyrénées-Atlantiques and linked to bird feeding practices [16] (Fig. 1A). Additional records were displayed in the map of [33], with confirmed and suspected cases in Aube and in Finistère respectively in 2011; and with suspected cases in Jura, Orne and Yonne in 2012 (Fig. 1A). All these earlier reports attest to the presence of finch trichomonosis, from west to east across the central part of France from 2010 to 2012, also with one event in the south west in 2011. Based on the data available, it appears that these outbreaks usually involved around or less than 20 individuals and mainly occur during the spring and summer. However, there was also one possible event in winter 2007/2008. Contrasting with the clear seasonality described in the UK, the disease remains apparently sporadic in France with discontinuous spatiotemporal occurrences.

In northern France, a previous event in *C. chloris* mortality was reported in June 2009, in Pas-de-Calais but not from the Boulonnais and the cause remained undetermined with only chloralose intoxication ruled out [15]. The present report extends the geographic range of occurrence of finch trichomonosis to northern France and confirms that transmission during the winter is possible, as suspected in 2007/2008. The outbreak appeared multifocal with a major focus in the Boulonnais, but also four cases recorded inland as far as near the Belgium border. Mainly based on data recorded by the bird ringers who monitor their ringing sites, the present study was able to identify and characterize the causative pathogen; however, it clearly represents a tiny fragment of the actual situation and numbers of diseased and dead birds must have been much higher.

Interestingly, in western France, similar reports of dead and diseased *C. chloris* were recorded in over one hundred areas across Brittany in Ille-et-Vilaine, Côtes-d’Armor, Finistère and Morbihan, at the same time [53] (Fig. 1A). There, a specific survey conducted among a network of participants in winter counting of garden birds returned 293 testimonies totalizing 718 dead birds (the majority were fringillids: 661 *C. chloris*, 25 *F. coelebs* and 13 *C. carduelis*). For this event, no typical clinical signs were recorded and the laboratory tests did not identify any cause since the only three carcasses sent for analysis were too degraded [53]. Therefore, no cause was clearly identified and only a list of potential causes affecting wild garden birds including trichomonosis, salmonellosis, avian pox and mycotoxin which can all be spread at feeders for garden birds were mentioned [53]. Based on the present study that confirmed an outbreak of finch trichomonosis in the north of France, if one considers the concurrent mortality recorded in Brittany possibly caused by *T. gallinae*, it can be speculated that the outbreak of finch trichomonosis during winter 2016/2017 was massive. It may have constituted the largest finch trichomonosis outbreak detected in France and was widespread geographically, possibly including the Normandy region located in between both affected regions. However, local supporting data are lacking.

### Surveillance of finch trichomonosis in France

The SAGIR network has contributed to the majority of the data related to finch trichomonosis in France [14–16]. The potential role of wildlife rescue centres for wild bird disease surveillance, including finch trichomonosis, has been highlighted previously [33]. Here, the role of bird ringers in the early detection and monitoring of the finch trichomonosis outbreak is emphasized. In an overall effort at the national level to survey this emerging disease with a deleterious impact [48], and that is rapidly spreading in Europe [27, 44, 46, 47, 61, 65, 70, 83] and France ([14–16, 32, 33, 53], present study), the CRBPO, the SAGIR, the Veterinary school of Nantes and some wildlife rescue centres launched a joint survey for finch trichomonosis and tit poxvirus in 2018. Relying on the capacities of these different bodies together, it will become more feasible to monitor the situation for these avian diseases in a timely and evenly spaced manner across the country. Regarding the Nord-Pas-de-Calais, fortunately, it has an efficient SAGIR network, a running wildlife rescue centre and is among the most covered regions by bird ringers. It will therefore be possible to pursue monitoring of the local situation in the long term. Preliminary data show no recurrence of similar massive outbreaks during the following years. Additionally, initiatives toward the public such as specific survey (e.g., the one conducted in Brittany) or dedicated online notification portal (e.g., BTO – Garden Wildlife Health) could provide valuable data for avian disease surveillance and should be considered.

### To ring or not to ring?

Bird ringers from the Cap-Ornis Association were the first to record and notify the SAGIR network about finch mortality in the Boulonnais. It is always a dilemma to decide to ring or not to ring a sickened individual. On the one hand, since the security of the bird comes first, and the ringing action implies unnecessary stress for the weaken bird that might have an additional negative impact on its survival, it could be advised not to ring. On the other, individualising diseased birds enables potential return of valuable information if the bird is recovered dead or alive (e.g., survival rate, survival time, dispersion and distance covered) to study the impact of diseases and the progress of epidemics. In the present study, it was decided to ring diseased birds and only record their weight to limit their handling and associated stress. The recovery rate of ringed diseased birds found dead was 35.3% (6/17), which is twenty fold higher than the average recovery rate of dead European greenfinches (all causes of death) in France 1.77% (2247/126,521, period 1910–2017, [18]). While small, these numbers clearly highlight the negative impact of trichomonosis in affected finch populations and provide some justification for the ringing of diseased individuals. Although the question remains debatable, a practical consensus must be strictly applied in the field when handling sick individuals, with the isolation of the bird and the application of good hygiene practices (such as use of disposable materials and equipment or their disinfection prior to new usage) in order to avoid the spread of pathogens through ringing activity.

### Natural introduction and anthropogenic spread of diseases

Although *T. gallinae* is considered to be fragile in the environment [79], its indirect transmission from food and water contaminated by the saliva of infected birds has long been hypothesized [2, 13, 16, 38, 39]. McBurney et al. [54] have experimentally demonstrated the persistence of *T. gallinae* in moist birdseeds with organic debris for up to 48 h, confirming that garden feeders in people’s gardens are a favourable place for transmission of the disease [54]. Readily available food and water sources regularly provided by humans attract many species of songbirds and particularly the European greenfinch [53] as well as the columbids (natural reservoirs of the parasite), and artificially increase the chances of inter-specific *T. gallinae* transmission [14]. Without any record of dead or diseased birds in the local populations in the autumn, it can be hypothesized that the suddenness of the outbreak is linked to introduction of the parasite in the finch populations wintering in the Boulonnais. The introduction of the disease could possibly originate from naturally infected columbids (known to be present in the Pas-de-Calais [15]) or from migrant birds and particularly finches originating from infected populations (e.g., Common chaffinches as suggested by [46]), gathering together at the same feeders. The rare presence of columbid at some of the studied ringing sites makes the first possibility less likely. However, the recapture of European greenfinches and Common chaffinches ringed in the UK and Fennoscandia in the Boulonnais, including at some of the affected ringing sites studied here, supports the second hypothesis [18]). Subsequently, the disease has spread locally, along with local bird movements and their usage of feeders within the area, leading to local foci. This idea is supported by the information from the diseased ringed birds found dead, that showed a short dispersion distance (0–4 km) and a survival time for up to five days, during which they eat desperately and keep regurgitating contaminated birdseeds at the feeders.

### Bird feeding practices

In France, bird feeding in the garden of private homes is a popular activity, essentially carried out during the winter when the weather conditions are unfavourable for birds. While wild avifauna can benefit from the supplementary food sources offered at the feeding stations, the anthropogenic feeding of birds also plays a potential role in spreading diseases among garden birds [2, 16, 38, 41, 47, 48, 54]. To remain beneficial, basic hygiene should be carefully maintained to prevent the risk of pathogen transmission. Simple steps such as the selection of feeders that prevent the food from becoming moist; the minimal provisioning of feeders with frequent replenishment; the removal of food waste and faeces; the dispersion and rotation of the different types of food and food stations at different locations are recommended [47], along with cleaning and disinfection of the feeding and water stations with water and soap or diluted bleach (according to the manufacturer’s recommendations) [22]. If the presence of diseased birds is noted at the feeders, all the above mentioned actions should be strictly and diligently followed to prevent the risk of pathogen transmission. In the event of outbreak, a temporary cessation of food provisioning might be considered but this action remains debated. Indeed, on the one hand suddenly stopping supplementary feeding will be detrimental for the all the birds relying on the given food point and will also likely induce a dispersion of the birds to other nearby feeders with a risk of promoting the spread of the disease geographically. On the other, continuing the supplementary feeding runs the risk of continued exposure and sustained transmission to finches and other passerine species visiting the affected feeding station. To mitigate the situation, it could be envisaged to slowly reduce provisioning. This type of approach could reduce the number of birds visiting the affected feeding point. Healthy individuals could disperse to find an alternate food point, reducing density and contact with infected birds that will likely continue to rely on the same food station. This will reduce local transmission and allow for easier control of the outbreak with strict hygiene actions.

## Supplementary materials

Supplementary material is available at https://www.parasite-journal.org/10.1051/parasite/2019022/olm
*Supplementary File*: Sequences of *Trichomonas gallinae* deposited in GenBank for all four genetic targets. *Supplementary File (Video)*: Trichomonasis symptoms in a European greenfinch (*Chloris chloris).*

## References

[1] Altschul SF, Gish W, Miller W, Myers EW, Lipman DJ. 1990 Basic local alignment search tool. Journal of Molecular Biology, 215, 403–410. 2231712 10.1016/S0022-2836(05)80360-2

[2] Anderson NL, Grahn RA, Van Hoosear K, Bondurant RH. 2009 Studies of trichomonad protozoa in free ranging songbirds: Prevalence of *Trichomonas gallinae* in house finches (*Carpodacus mexicanus*) and corvids and a novel trichomonad in mockingbirds (*Mimus polyglottos*). Veterinary Parasitology, 161, 178–186. 19278788 10.1016/j.vetpar.2009.01.023

[3] ANSES. 2017 Détection de génome de virus influenza aviaire de sous-type H5 de la lignée eurasienne selon la méthode de RT-PCR temps réel gène H5-HA2 avec témoin positif « non cible » externe – rRT-PCR AIV H5-HA2 avec IPC-M. Agence National de Sécurité Sanitaire de l’Alimentation, de l’Environnement et du Travail: France.

[4] Baker JR. 1986 Trichomoniasis, a major cause of vomiting in budgerigars. Veterinary Record, 118, 447–449. 3716106 10.1136/vr.118.16.447

[5] Bigland GH. 1966 Common diseases of noncommercial and pet birds. Canadian Veterinary Journal, 7, 252–259. 6009950 PMC1696656

[6] Boal CW, Mannan RW, Hudelson KS. 1998 Trichomoniasis in Cooper’s hawks from Arizona. Journal of Wildlife Diseases, 34, 590–593. 9706569 10.7589/0090-3558-34.3.590

[7] Cepicka I, Kutišová K, Tachezy J, Kulda J, Flegr J. 2005 Cryptic species within *Tetratrichomonas gallinarum* species complex revealed by molecular polymorphism. Veterinary Parasitology, 128, 11–21. 15725528 10.1016/j.vetpar.2004.11.003

[8] Chalmers GA. 1992 Trichomoniasis in finches. Canadian Veterinary Journal, 33, 616–617. PMC1481342 17424080

[9] Chavatte J-M, Okumura C, Landau I. 2017 Redescription of *Babesia ardeae* Toumanoff, 1940, a parasite of Ardeidae, including molecular characterization. Parasitology Research, 116, 1089–1097. 28160075 10.1007/s00436-017-5394-1

[10] Chi JF, Lawson B, Durrant C, Beckmann K, John S, Alrefaei AF, Kirkbride K, Bell DJ, Cunningham AA, Tyler KM. 2013 The finch epidemic strain of *Trichomonas gallinae* is predominant in British non-passerines. Parasitology, 140, 1234–1245. 23920088 10.1017/S0031182013000930

[11] Cole RA. 1999 Trichomoniasis, in Field Manual of Wildlife Diseases: General Field Procedures and Diseases of Birds. Friend M, Franson JC, Editors. U.S. Department of Interior, U.S. Geographical Survey: Washington, D.C, USA p. 201–206.

[12] Cooper JE, Petty SJ. 1988 Trichomoniasis in free living goshawks (*Accipiter gentilis*) from Great Britain. Journal of Wildlife Diseases, 24, 80–87. 3352101 10.7589/0090-3558-24.1.80

[13] Cousquer G. 2005 Ingluvitis and oesophagitis in wild finches. Veterinary Record, 157, 455. 10.1136/vr.157.15.455-a 16215253

[14] Décors A, Mastain O. 2010 Épidémiosurveillance de la faune sauvage – Bilan des analyses effectuées de 2006 à 2008 dans le cadre du Réseau SAGIR, réseau ONCFS/FNC/FDC. Paris: Office National de la Chasse et de la Faune Sauvage.

[15] Décors A, Moinet M, Mastain O. 2011 SAGIR – Bilan 2009–2010. Paris: Office National de la Chasse et de la Faune Sauvage.

[16] Décors A, Lesage C, Moinet M. 2013 Réseau SAGIR – Bilan 2011. Paris: Office National de la Chasse et de la Faune Sauvage.

[17] Décors A, Hars J, Faure R, Quintaine T, Chollet J-Y, Rossi S. 2015 Le réseau SAGIR : un outil vis-à-vis des agents pathogènes exotiques. Bulletin Epidémiologique – Santé Animale et Alimentation, 66, 35–39.

[18] Dehorter O, CRBPO. 2018 Bird ringing and movement database for France. Centre de Recherches sur la Biologie des Populations d’Oiseaux. Paris, France: Muséum National d’Histoire Naturelle http://crbpo.mnhn.fr/. Accessed on 01/07/2018.

[19] Duff JP, Holmes P, Brown I, Gayford P. 2003 Surveillance scheme for wildlife disease in England and Wales. Veterinary Record, 153, 538. 14620559

[20] Ecco R, Preis IS, Vilela DA, Luppi MM, Malta MC, Beckstead RB, Stimmelmayr R, Gerhold RW. 2012 Molecular confirmation of *Trichomonas gallinae* and other parabasalids from Brazil using the 5.8S and ITS-1 rRNA regions. Veterinary Parasitology, 190, 36–42. 22749289 10.1016/j.vetpar.2012.05.029

[21] Euzéby J. 1982 Diagnostic expérimental des helminthoses animales – Livre 2: « Diagnostic post mortem et diagnostic immunologique ». Paris: Informations Techniques des Services Vétérinaires.

[22] Feliciano LM, Underwood TJ, Aruscavage DF. 2018 The effectiveness of bird feeder cleaning methods with and without debris. Wilson Journal of Ornithology, 130, 313–320.

[23] Felleisen RS. 1997 Comparative sequence analysis of 5.8S rRNA genes and internal transcribed spacer regions (ITS) of trichomonadid protozoa. Parasitology, 115, 111–119. 10190167 10.1017/s0031182097001212

[24] Forrester DJ, Foster GW. 2008 Trichomonosis, in Parasitic diseases of wild birds. Atkinson CT, Thomas NJ, Hunter DB, Editors. Wiley-Blackwell: Ames, Iowa p. 120–153.

[25] Forzán MJ. 2009 Trichomoniasis in wild finches. Canadian Veterinary Journal, 50, 561. 19721776 PMC2684042

[26] Forzán MJ, Vanderstichel R, Melekhovets YF, McBurney S. 2010 Trichomoniasis in finches from the Canadian maritime provinces: an emerging disease. Canadian Veterinary Journal, 51, 391–396. 20592828 PMC2839828

[27] Ganas P, Jaskulska B, Lawson B, Zadravec M, Hess M, Bilic I. 2014 Multi-locus sequence typing confirms the clonality of *Trichomonas gallinae* isolates circulating in European finches. Parasitology, 141, 652–661. 24476813 10.1017/S0031182013002023

[28] Gauthier D, Lamberger K, Décors A. 2016 Vade-mecum des laboratoires départementaux d’analyses vétérinaires – Le diagnostic des maladies de la faune sauvage libre. Office National de la Chasse et de la Faune Sauvage: Paris.

[29] Gerhold RW, Yabsley MJ, Smith AJ, Ostergaard E, Mannan W, Cann JD, Fischer JR. 2008 Molecular characterization of the *Trichomonas gallinae* morphologic complex in the United States. Journal of Parasitology, 94, 1335–1341. 18576862 10.1645/GE-1585.1

[30] Girard YA, Rogers KH, Gerhold R, Land KM, Lenaghan SC, Woods LW, Haberkern N, Hopper M, Cann JD, Johnsona CK. 2014 *Trichomonas stableri* n. sp, an agent of trichomonosis in Pacific Coast band-tailed pigeons (*Patagioenas fasciata monilis*). International Journal for Parasitology – Parasites & Wildlife, 3, 32–40. 24918075 10.1016/j.ijppaw.2013.12.002 PMC4047957

[31] Girard YA, Rogers KH, Woods LW, Chouicha N, Miller WA, Johnson CK. 2014 Dual-pathogen etiology of avian trichomonosis in a declining band-tailed pigeon population. Infection, Genetics and Evolution, 24, 146–156. 10.1016/j.meegid.2014.03.002 24632451

[32] Gourlay P, Décors A, Jouet D, Treilles D, Lemberger K, Faure E, Moinet M, Chi J, Tyler K, Cunningham A, Lawson B. 2011 Finch trichomonosis spreads to France. European Section of the Wildlife Disease Association Bulletin, 2, 9–10.

[33] Gourlay P, Décors A, Moinet M, Lambert O, Lawson B, Beaudeau F, Assié S. 2014 The potential capacity of French wildlife rescue centres for wild bird disease surveillance. European Journal of Wildlife Research, 60, 865–873.

[34] Grabensteiner E, Bilic I, Kolbe T, Hess M. 2010 Molecular analysis of clonal trichomonad isolates indicate the existence of heterogenic species present in different birds and within the same host. Veterinary Parasitology, 172, 53–64. 20471174 10.1016/j.vetpar.2010.04.015

[35] Haugen AO. 1952 Trichonomiasis in Alabama mourning doves. Journal of Wildlife Management, 16, 164–169.

[36] Henry P-Y, CRBPO. 2016 Protocol SPOL MANGEOIRE, v2.6. Centre de Recherches sur la Biologie des Populations d’Oiseaux. Paris, France: Muséum National d’Histoire Naturelle.

[37] Höfle U, Blanco JM, Palma L, Melo P. 2000 Trichomoniasis in Bonelli’s eagle (*Hieraaetus fasciatus*) nestlings in south-west Portugal, in Zoological Education. Raptor Biomedicine III, Lumej JT, Remple JD, Redig PT, Lierz M, Cooper JE, Editors. FL Network Inc: Lake Worth p. 45–52.

[38] Höfle U, Gortázar C, Ortíz JA, Knispel B. 2004 Outbreak of Trichomoniasis in a woodpigeon wintering roost. European Journal of Wildlife Research, 50, 73–77.

[39] Holmes P, Duff P. 2005 Ingluvitis and oesophagitis in wild finches. Veterinary Record, 157, 455. 10.1136/vr.157.15.455 16215254

[40] Keymer IF. 1972 Diseases of birds of prey. Veterinary Record, 90, 579–594. 5073130 10.1136/vr.90.21.579

[41] Kocan RM. 1969 Various grains and liquid as potential vehicles of transmission for *Trichomonas gallinae*. Journal of Wildlife Diseases, 5, 148–149. 10.7589/0090-3558-5.3.148 5817771

[42] Krone O, Altenkamp R, Kenntner N. 2005 Prevalence of *Trichomonas gallinae* in northern goshawks from the Berlin area of northeastern Germany. Journal of Wildlife Diseases, 41, 304–309. 16107664 10.7589/0090-3558-41.2.304

[43] Larkin MA, Blackshields G, Brown NP, Chenna R, McGettigan PA, McWilliam H, Valentin F, Wallace IM, Wilm A, Lopez R, Thompson JD, Gibson TJ, Higgins DG. 2007 Clustal W and Clustal X version 2.0. Bioinformatics, 23, 2947–2948. 17846036 10.1093/bioinformatics/btm404

[44] Lawson B, Cunningham AA, Chantrey J, Hughes LA, Kirkwood J, Pennycott T, Simpson V. 2006 Epidemic finch mortality. Veterinary Record, 159, 367. 10.1136/vr.159.11.367-a 16963722

[45] Lawson B, Cunningham AA, Chantrey J, Hughes LA, John SK, Bunburry N, Bell DJ, Tyler KM. 2011 A clonal strain of *Trichomonas gallinae* is the aetiologic agent of an emerging avian epidemic diseases. Infection, Genetics and Evolution, 11, 1638–1645. 10.1016/j.meegid.2011.06.007 21712099

[46] Lawson B, Robinson RA, Neimanis A, Handland K, Isomursu M, Agren EO, Hamnes IS, Tyler KM, Chantrey J, Hugnes LA, Pennycott TW, Simpson VR, John SK, Peck KM, Toms MP, Bennett M, Kirkwood JK, Cunningham AA. 2011 Evidence of spread of the emerging infectious disease finch trichomonosis, by migrating birds. Ecohealth, 42, 143–153. 10.1007/s10393-011-0696-8 21935745

[47] Lawson B, Robinson RA, Colvile KM, Peck KM, Chantrey J, Pennycott TW. 2012 The emergence and spread of finch trichomonosis in the British Isles. Philosophical Transactions of the Royal Society B, 367, 2852–2863. 10.1098/rstb.2012.0130 PMC3427565 22966140

[48] Lawson B, Robinson RA, Toms MP, Risely K, MacDonald S, Cunningham AA. 2018 Health hazards to wild birds and risk factors associated with anthropogenic food provisioning. Philosophical Transactions of the Royal Society B, 373, 20170091. 10.1098/rstb.2017.0091 PMC5882997 29531146

[49] Lecollinet S, Blanchard Y, Manson C, Lowenski S, Laloy E, Quenault H, Touzain F, Lucas P, Eraud C, Bahuon C, Zientara S, Beck C, Décors A. 2015 Dual Emergence of Usutu Virus in Common Blackbirds, Eastern France. Emerging Infectious Diseases, 22, 2225–2227. 10.3201/eid2212.161272 PMC5189168 27869608

[50] Lehikoinen A, Lehikoinen E, Valkama J, Väisänen RA, Isomursu M. 2013 Impacts of trichomonosis epidemics on Greenfinch *Chloris chloris* and Chaffinch *Fringilla coelebs* populations in Finland. Ibis, 155, 357–366.

[51] Martínez-Díaz R, Ponce-Gordo F, Rodríguez-Arce I, del Martínez-Herrero MC, González FG, Molina-López RÁ, Gómez-Muñoz MT. 2015 *Trichomonas gypaetinii* n. sp, a new trichomonad from the upper gastrointestinal tract of scavenging birds of prey. Parasitology Research, 114, 101–112. 25273632 10.1007/s00436-014-4165-5

[52] Marx M, Reiner G, Willems H, Rocha G, Hillerich K, Masello JF, Mayr SL, Moussa S, Dunn JC, Thomas RC, Goodman SJ, Hamer KC, Metzger B, Cecere JG, Spina F, Koschkar S, Calderón L, Romeike T, Quillfeldt P. 2017 High prevalence of *Trichomonas gallinae* in wild columbids across western and southern Europe. Parasites & Vectors, 10, 242. 28521843 10.1186/s13071-017-2170-0 PMC5437606

[53] Mathérion D, Février Y, Nègre I. 2017 Mortalité de verdier d’Europe (Carduelis chloris – Greenfinch) en Bretagne durant l’hiver 2016–2017. Le Râle d’eau, 171, 7–10.

[54] McBurney S, Kelly-Clark WK, Forzán MJ, Vanderstichel R, Teather K, Greenwood SJ. 2017 Persistence of *Trichomonas gallinae* in Birdseed. Avian Diseases, 61, 311–315. 28956991 10.1637/11545-113016-RegR1

[55] McKeon T, Dunsmore J, Raida SR. 1997 *Trichomonas gallinae* in budgerigars and columbid birds in Perth, Western Australia. Australian Veterinary Journal, 75, 652–655. 9325543 10.1111/j.1751-0813.1997.tb15363.x

[56] Medlin L, Elwood HJ, Sickel S, Sogin ML. 1988 The characterization of enzymatically amplified eukaryotic 16S-like rRNA-coding regions. Gene, 71, 491–499. 3224833 10.1016/0378-1119(88)90066-2

[57] Mehlhorn H, Al-Quraishy S, Aziza A, Hess M. 2009 Fine structure of the bird parasites *Trichomonas gallinae* and *Tetratrichomonas gallinarum* from cultures. Parasitology Research, 105, 751–756. 19421777 10.1007/s00436-009-1451-8

[58] Mesa CP, Stabler RM, Berthrong M. 1961 Histopathological changes in the domestic pigeon infected with *Trichomonas gallinae* (Jones-Barn strain). Avian Diseases, 5, 48–60.

[59] Mirzaei M, Ghashghaei O, Khedri J. 2016 First report of an outbreak trichomoniasis in turkey in Sistan, Iran. Journal of Parasitic Diseases, 40, 61–64. 27065599 10.1007/s12639-014-0445-3 PMC4815854

[60] Narcisi EM, Sevoian M, Honigberg BN. 1991 Pathologic changes in pigeons infected with a virulent *Trichomonas gallinae* strain (Eiberg). Avian Diseases, 35, 55–61. 2029262

[61] Neimanis AS, Handeland K, Isomursu M, Agren E, Mattsson R, Hamnes IS, Bergsjø B, Hirvelä-Koski V. 2010 First report of epizootic trichomoniasis in wild finches (Family Fringillidae) in Southern Fennoscandia. Avian Diseases, 54, 136–141. 20408413 10.1637/8952-060509-Case.1

[62] NFU47101-AFNOR. 2007 Isolement et identification de tout serovar ou de serovar(s) spécifié(s) de salmonelles chez les oiseaux. La Plaine Saint-Denis: Association Française de Normalisation.

[63] Pennycott TW. 1998 Carriage of trichomonads, *Hexamita* species and *Blastocystis* species by adult pheasants. Veterinary Record, 143, 142–143. 9725187 10.1136/vr.143.5.142

[64] Pennycott TW, Lawson B, Cunningham AA, Simpson V, Chantrey J. 2005 Necrotic ingluvitis in wild finches. Veterinary Record, 157, 360. 16170010 10.1136/vr.157.12.360

[65] Peters M, Kilwinski J, Reckling D, Henning K. 2009 Gehäufte todesfälle von wild lebended grünfinken an futterstellen infolge *Trichomonas gallinae* infektionen — ein aktuelles problem in Norddeutschland. Kleintierpraxis, 54, 433–438.

[66] Ponce-Gordo F, Herrera S, Castro AT, Garcia-Duran B, Martinez-Diaz RA. 2002 Parasites from farmed ostriches *Struthio camelus* and rheas *Rhea americana* in Europe. Veterinary Parasitology, 107, 137–160. 12072221 10.1016/s0304-4017(02)00104-8

[67] Real J, Mañosa S, Muñoz E. 2000 Trichomoniasis in a Bonelli’s eagle population in Spain. Journal of Wildlife Diseases, 36, 64–70. 10682745 10.7589/0090-3558-36.1.64

[68] Réseau SAGIR. 2008 Surveillance sanitaire de la faune sauvage en France. Lettre n 162 Paris: Office National de la Chasse et de la Faune Sauvage.

[69] Réseau SAGIR. 2011 Programme de surveillance de la trichomonose avaiaire: Avril – Octobre 2011 – Impact de la trichomonose sur le succès reproducteur des pigeons colombins. Paris: Office National de la Chasse et de la Faune Sauvage.

[70] Robinson RA, Lawson B, Toms MP, Peck KM, Kirkwood JK, Chantrey J, Clatworthy IR, Evans AD, Hughes LA, Hutchinson OC, John SK, Pennycott TW, Perkins MW, Rowley PS, Simpson VR, Tyler KM, Cunningham AA. 2010 Emerging infectious disease leads to rapid population declines of common British birds. PLoS One, 5, e12215. 20805869 10.1371/journal.pone.0012215 PMC2923595

[71] Samour JH, Bailey TA, Cooper JE. 1995 Trichomoniasis in birds of prey (order Falconiformes) in Bahrain. Veterinary Record, 136, 358–362. 7610541 10.1136/vr.136.14.358

[72] Sansano-Maestre J, Garijo-Toledo MM, Gómez-Munoz MT. 2009 Prevalence and genotyping of *Trichomonas gallinae* in pigeons and birds of prey. Avian Pathology, 38, 201–207. 19468936 10.1080/03079450902912135

[73] Schwarz GE. 1978 Estimating the dimension of a model. Annals of Statistics, 6, 461–464.

[74] Stabler RM. 1947 *Trichomonas gallinae*, pathogenic trichomonad of birds. Journal of Parasitology, 33, 207–210. 20245737

[75] Stabler RM. 1947 Strains of *Trichomonas gallinae* varying in virulence. Journal of Parasitology, 33, 8. 20340855

[76] Stabler RM. 1948 Variations in virulence of *Trichomonas gallinae* in pigeons. Journal of Parasitology, 34, 147–149. 18856299

[77] Stabler RM. 1953 Frounce: Its cause and cure. Falconry News and Notes, 1, 4–5.

[78] Stabler RM. 1953 Effect of *Trichomonas gallinae* (Protozoa; Mastigophora) on nestling passerine birds. Journal of the Colorado-Wyoming Academy of Science, 4, 58.

[79] Stabler RM. 1954 *Trichomonas gallinae*: a review. Experimental Parasitology, 3, 368–402. 13183096 10.1016/0014-4894(54)90035-1

[80] Tamura K. 1992 Estimation of the number of nucleotide substitutions when there are strong transition-transversion and G+C-content biases. Molecular Biology and Evolution, 9, 678–687. 1630306 10.1093/oxfordjournals.molbev.a040752

[81] Villanúa D, Höfle U, Pérez-Rodríguez L, Gortázar C. 2006 *Trichomonas gallinae* in wintering Common Wood Pigeons *Columba palumbus* in Spain. Ibis, 148, 641–648.

[82] Wang Y, Tian RM, Gao ZM, Bougouffa S, Qian P-Y. 2014 Optimal eukaryotic 18S and universal 16S/18S ribosomal RNA primers and their application in a study of symbiosis. PLoS One, 9, e90053. 24594623 10.1371/journal.pone.0090053 PMC3940700

[83] Zadravec M, Marhold C, Slavec B, Zorman Rojs O, Racnik J, Gruntar I. 2012 Trichomonosis in finches in Slovenia. Veterinary Record, 171, 253–254. 22961796 10.1136/vr.e5973

