# Supplementary material for: An outbreak of trichomonosis in European greenfinches Chloris chloris and European goldfinches Carduelis carduelis wintering in Northern France
Source: Parasite. 2019 Apr 8;26:21. doi: 10.1051/parasite/2019022 (PMC6452646; doi:10.1051/parasite/2019022)
Supplement: Supplementary file 1 — Supplementary File: Sequences of Trichomonas gallinae deposited in GenBank for all four genetic targets. [file parasite-26-21-s1.pdf]

Appendix 1

Identity	Sequence (Acc. No.)	Hosts	Country	Reference
ITS region (MK172843-MK172847; position: 1554-1924)				
100%	AY349182	Pigeon	Brazil	Kleina et al., 2004, Int J Parasitol, 34(8):963-70
	EF208019	*Nesoenas mayeri*<br>*Streptopelia picturata*	Mauritius	Gaspar da Silva et al., 2007 Infect Genet Evol,7(4):433-40
	EU215369	*Patagioenas fasciata*<br>*Accipiter cooperii*<br>*Haemorhous mexicanus*<br>*Zenaida macroura*<br>*Columba livia*	USA	Gerhold et al., 2008, J Parasitol, 94(6):1335-41
	EU290649	*Haemorhous mexicanus*<br>*Aphelocoma californica*<br>*Corvus brachyrhynchos*	USA	Anderson et al., 2009, Vet Parasitol, 161(3-4):178-86
	EU881911<br>EU881913<br>EU881915<br>EU881916	*Columba livia*<br>*Tyto alba*<br>*Hieraaetus fasciatus*<br>*Falco tinnunculus*	Spain	Sansano-Maestre et al., 2009, Avian Pathol, 38(3):201-7
	FN433476	*Columba livia*	Austria	Grabensteiner et al., 2010, Vet Parasitol, 172(1-2):53-64
	GQ150752	*Chloris chloris*	UK	Robinson et al., 2010, PLoS One, 5(8):e12215
	HG008050	*Chloris chloris*	Austria	Ganas et al., 2014, Parasitology, 141(5):652-61
	JN007005	*Melopsittacus undulatus*	Austria	Reinmann et al., 2012, Vet Parasitol, 185(2-4):138-44
	JQ755279-JQ755283	Columbids	Australia	unpublished
	JX089398	*Streptopelia decaocto*	Saint Kitts & Nevis	Ecco et al., 2012, Vet Parasitol, 190(1-2):36-42
	KC215387	*Patagioenas fasciata*	USA	Girard et al., 2014, Int J Parasitol Parasites Wildl, 3(1):32-40
	KF214772	*Spinus tristis*	USA	McBurney et al., 2015, Parasitology, 142(8):1053-62
	KF993693<br>KF993694	*Pica pica*<br>*Streptopelia decaocto*	Spain	Martínez-Diáz et al., 2015, Parasitol Res, 114(1):101-12

	KF993695	*Melopsittacus undulatus*		
	KF993696	*Bubo bubo*		
	KF993697	*Falco tinnunculus*		
	KF993698	*Tyto alba*		
	KF993699	*Hieraaetus pennatus*		
	KF993700	*Accipiter gentilis*		
	KF993701	*Buteo buteo*		
	KF993702	*Circus cyaneus*		
	KF993703	*Strix aluco*		
	KF993704	*Asio otus*		
	KJ721785	*Columba livia*	China	Jiang et al., 2016, Vet J, 211:88-93
	KJ776739	*Accipiter nisus*	Spain	Martínez-Herrero et al., 2014, Avian Pathol, 43(6):535-46
	KJ776741	*Falco tinnunculus*		
	KJ776742	*Otus scops*		
	KM095107-M095108	*Accipiter nisus*	Czech Republic	Kunca et al., 2015, Folia Parasitol, 62, pii:2015.035
	KT003194	*Tyto alba*	USA	unpublished
	KU954107	*Columba livia*	Poland	Bobrek et al., 2017, Pol J Vet Sci, 20(1):185-187
	KX459440-KX459444	*Columba oenas*	Germany	Marx et al., 2017, Parasit Vectors, 10(1):242
	KX514378	*Bubo bubo*	Spain	Martínez-Herrero et al., 2017, Infect Genet Evol, 55:93-103
	KX584000	*Serinus canaria*	Slovenia	Zadravec et al., 2017, Vet Parasitol, 239:90-93
	KX844990- KX844991	*Streptopelia decaocto*	Malta	Marx et al 2017, Parasit Vectors, 10(1):242
18S ssrRNA gene (MK172843-MK172847; position: 1-1581)				
100%	EU215375	*Buteo platypterus*	USA	Gerhold et al., 2008, J Parasitol, 94(6):1335-41
	FN433484	*Melopsittacus undulatus*	Austria	Grabensteiner et al., 2010, Vet Parasitol, 172(1-2):53-64
	HG008106	*Chloris chloris*	Austria	Ganas et al., 2014, Parasitology, 141(5):652-61
	KM095107	*Accipiter nisus*	Czech Republic	Kunca et al., 2015, Folia Parasitol, 62, pii:2015.035
	KM246603	*Circus cyaneus*	Spain	Martínez-Diáz et al., 2015, Parasitol Res, 114(1):101-12
	KM246604	*Falco tinnunculus*		
	KM246605	*Pica pica*		
	KM246606	*Bubo bubo*		

	KU954105	*Columba livia*	Poland	Bobrek et al., 2017, Pol J Vet Sci, 20(1):185-187
	KX514379	*Bubo bubo*	Spain	Martínez-Herrero et al., 2017, Infect Genet Evol, 55:93-103
	KX584001	*Serinus canaria*	Slovenia	Zadravec et al., 2017, Vet Parasitol, 239:90-93
hdg gene (MK172848 to MK172852)				
100%	HG008114	*Chloris chloris*	Austria	Ganas et al., 2014, Parasitology, 141(5):652-61
	JF681136	*Chloris chloris*	UK	Lawson et al., 2011, Infect Genet Evol, 11(7):1638-45
	KC244201	*Patagioenas fasciata*	USA	Girard et al., 2014, Infect Genet Evol, 24:146-56
	KP900023	*Accipiter gentilis*	Spain	Sansano-Maestre et al., 2016, Res Vet Sci, 107:182-189
	KP900024	*Aquila fasciata*		
	KP900025	*Bubo bubo*		
	KP900026	*Columba livia*		
	KP900027	*Falco tinnunculus*		
	KP900028	*Strix aluco*		
	KP900029	*Tyto alba*		
	KX514380	*Bubo bubo*		Martínez-Herrero et al., 2017, Infect Genet Evol, 55:93-103
	KX584002	*Serinus canaria*	Slovenia	Zadravec et al., 2017, Vet Parasitol, 239:90-93
rpb1 gene (MK172853 to MK172857)				
100%	HG008113	*Chloris chloris*	Austria	Ganas et al., 2014, Parasitology, 141(5):652-61
	HM016230	*Accipiter cooperii*	USA	Malik et al., 2011, PLoS One, 6(6):e20774
	KF233589	*Patagioenas fasciata*	USA	Girard et al., 2014, Int J Parasitol Parasites Wildl, 3(1):32-40
